# The housing first technical assistance and training (HFTAT) implementation strategy: outcomes from a mixed methods study of three programs

**DOI:** 10.1186/s13011-018-0172-3

**Published:** 2018-09-21

**Authors:** Dennis P. Watson, Emily Q. Ahonen, Valery Shuman, Molly Brown, Sam Tsemberis, Philip Huynh, Fangqian Ouyang, Huiping Xu

**Affiliations:** 1 Center for Dissemination and Implementation Science, University of Illinois College of Medicine at Chicago, 1603 W Taylor St, Chicago, IL 60612 USA; 2 Indiana University Richard M. Fairbanks School of Public Health, 1050 Wishard Blvd, Indianapolis, IN 46202 USA; 3 Heartland Alliance Health, Midwest Harm Reduction Institute, 1207 W. Leland Ave, Chicago, IL 60640 USA; 4 Department of Psychology, DePaul University, 1 E. Jackson, Chicago, IL 60604 USA; 5 Department of Psychiatry, NYPH, Columbia University Medical Center, 1051 Riverside Drive, New York, NY 10032 USA; 6 Indiana University School of Medicine, 340 W. 10th St, Indianapolis, IN 46202 USA

**Keywords:** Housing first, Harm reduction, Implementation, Implementation strategy, eLearning, Consultation, Storytelling, Community of practice, Digital badging

## Abstract

**Background:**

This paper discusses the initial testing of the Housing First Training and Technical Assistance (HFTAT) Program, a multifaceted, distance-based strategy for the implementation of the Housing First (HF) supportive housing model. HF is a complex housing intervention for serving people living with serious mental illness and a substance use disorder that requires significant individual- and structural-level changes to implement. As such, the HFTAT employs a combined training and consultation approach to target different levels of the organization. Training delivered to all organizational staff focuses on building individual knowledge and uses narrative storytelling to overcome attitudinal implementation barriers. Consultation seeks to build skills through technical assistance and fidelity audit and feedback.

**Method:**

We employed a mixed method design to understand both individual-level (e.g., satisfaction with the HFTAT, HF knowledge acquisition and retention, and HF acceptability and appropriateness) and structural-level (e.g., fidelity) outcomes. Quantitative data were collected at various time points, and qualitative data were collected at the end of HFTAT activities. Staff and administrators (*n* = 113) from three programs across three states participated in the study.

**Results:**

Satisfaction with both training and consultation was high, and discussions demonstrated both activities were necessary. Flexibility of training modality and narrative storytelling were particular strengths, while digital badging and the community of practice were perceived as less valuable because of incompatibilities with the work context. HF knowledge was high post training and retained after 3-month follow-up. Participants reported training helped them better understand the model. Attitudes toward evidence-based interventions improved over 6 months, with qualitative data supporting this but demonstrating some minor concerns related to acceptability and appropriateness. Fidelity scores for all programs improved over 9 months.

**Conclusion:**

The HFTAT was a well-liked and generally useful implementation strategy. Results support prior research pointing to the value of both (a) multifaceted strategies and (b) combined training and consultation approaches. The study also provides evidence for narrative storytelling as an approach for changing attitudinal implementation barriers. The need for compatibility between specific elements of an implementation strategy and the work environment was also observed.

**Electronic supplementary material:**

The online version of this article (10.1186/s13011-018-0172-3) contains supplementary material, which is available to authorized users.

## Background

Better understandings of strategies to integrate evidence-based interventions (EBIs) for behavioral health into routine practice are needed; however, theoretically based, well-described, and testable implementation strategies are few and far between [1–3]. Recent efforts to establish a common language for this line of inquiry have led to the identification and description of a wide range of discrete implementation strategies [2, 4], and additional literature demonstrates the need to elucidate the effectiveness of these strategies, either alone or in multifaceted or packaged combinations [5, 6]. This paper contributes to this growing area of study by discussing results from the testing of the Housing First Technical Assistance and Training Program (HFTAT), a multifaceted implementation strategy for the Housing First (HF) model.

### Overview of the HF model

HF, an EBI for housing chronically homeless individuals with dually diagnosed mental health and substance use disorders, is proven efficacious in relation to a wide variety of health and social outcomes [7–9]. There are a number of concerns related to HF model integrity in the United States. Most importantly, the U. S. Department of Housing and Urban Development has done little to enforce HF fidelity, with senior officials having advocated for housing continuums guided by a HF philosophy that emphasizes low-barrier placement for the most vulnerable without appropriate funding increases to provide additional needed services [10]. HF is a complex intervention requiring interaction between multiple levels within an organization, as well as the broader service system. Because of its widespread dissemination, the HF model requires adaptations to local contexts to be successful [11–15]. Furthermore, HF programs experience high levels of staff turnover that pose problems for consistency and sustainability of practice [1, 12, 16].

In HF, one of the more difficult practices to implement is harm reduction, an approach that works with clients at their own pace to mitigate negative consequences of substance use, rather than requiring a program-paced approach to abstinence. Harm reduction is an essential component of HF that complements its recovery-oriented service philosophy by focusing on a person’s overall well-being, as opposed to simply controlling behavioral health symptoms. Demonstrating the magnitude of HF’s harm reduction implementation problem, Watson et al. [17] found 18 out of 39 self-labeled ‘HF programs’ in a national sample required sobriety from clients after they were housed. Reasons harm reduction is often absent or inadequately implemented within HF programs include insufficient understanding of its centrality to the program and status quo resistance to harm reduction that exists within a service culture dominated by 12-step, abstinence-only programming [10, 18–21]. As such, implementation of effective harm reduction practice often requires shifting staff attitudes and values. Taking this into consideration, we sought to design an implementation strategy to address attitudinal barriers to the harm reduction component of HF, as well as improving (a) individual-level knowledge and skills and (b) structural-level policies and procedures.

### HFTAT component descriptions and logic underlying them

Previous literature has identified the need for implementation strategies to ensure the various organizational levels key to implementation success are addressed during the implementation process [6, 22, 23]. As such, we designed the HFTAT to be multifaceted (i.e., comprising various discrete components, each specifically targeting a specific implementation goal). We also designed it to be delivered over a distance using internet and communications technology to increase its geographical reach and lower its potential cost (compared to intensive face-to-face strategies), thus increasing the number of programs it can potentially serve [24, 25]. The HFTAT comprises two main components, (1) training and (2) consultation, which also include a number of additional nested components explained below. Additional details of the HFTAT not described here can be found in previously published articles [26, 27] and Additional file 1.

The HFTAT’s *training component* is aimed at impacting individual-level knowledge, skills, and attitudes, with different modules targeted to one or more groups of individuals within the organization (e.g., administrators/manager/supervisors, clinicians and case managers, staff without clinical responsibilities). It is delivered through *self-paced, asynchronous eLearning modules* to accommodate staff members’ often unpredictable work schedules and address likely organizational turnover. The eLearning modules incorporate features demonstrated to improve both adult learning and implementation outcomes such as *interactive activities* and *opportunities for reflection* on material learned [22, 28, 29] to overcome noted limitations of training for affecting individual-level behavior change necessary for implementation success [5, 22, 30–32]. We also embedded *narrative stories* from real HF tenants and staff to address attitudinal barriers to harm reduction. Storytelling has been demonstrated to be both an effective means of health communication and an approach for building tacit knowledge necessary for true and lasting attitude and behavior changes to occur [33–35]. Nested within the modules are links to an *online community of practice* (CoP) with access to *implementation resources* and incentives for eLearning completion in the form of *digital badges*. CoPs provide a space for participants to learn through social interactions with others engaged in similar work [36, 37]. Like storytelling, CoPs have been demonstrated to improve knowledge integration that can expand learning beyond simple skill building [38]. Virtual (i.e., online) CoPs are considered a flexible and affordable means of encouraging interaction between members [39]; however, there is relatively little evidence to date for their effectiveness in this capacity. The digital badges are an alternative online credentialing mechanism with potential to motivate eLearning participants [40].

The HFTAT’s *consultation* component begins after members of an organization’s identified implementation team have completed the eLearning modules. Training and consultation is a promising combination for improving implementation outcomes [1, 24, 41, 42]. For instance, Herschell et al. [43] found the combination of training and consultation provided to clinicians enhanced knowledge, skills, adherence, and client outcomes more consistently than training alone. The implementation team participating in the HFTAT’s consultation activities comprises administrators and implementation champions and is aimed at improving their HF leadership skills (strong leadership is an established component of implementation success [40, 44]) and affecting changes at the structural-level of the organization (i.e., policies and procedures). Consultation is performed by two HF experts (i.e., individuals with multiple years working in and providing training for HF programming), lasts approximately 6 months, and includes the following nested components: an *implementation manual*; *baseline assessment*; development of a *tailored implementation plan*; fidelity *audit and feedback*; and weekly *technical assistance* (TA) calls where the implementation team is encouraged to troubleshoot issues that arise during the implementation process—a major focus of which is how to handle situations using a harm reduction approach. Throughout consultation, HF experts continually assess implementation barriers and make HF adaptation recommendations based on the organization’s unique context.

In this paper, we explore how well the HFTAT performed in relation to training outcomes (e.g., satisfaction, knowledge gain and retention) and implementation outcomes (e.g., acceptability, appropriateness, and fidelity) within three organizations providing housing and services for formerly chronically homeless individuals. Where most previous research on training and consultation has focused largely on outcomes related to the individual clinician, we sought to understand the HFTAT’s ability to impact both individual- and structural-level outcomes. Furthermore, our use of narrative story telling fills another gap in the current literature, as clinician-level studies have largely focused on the transfer of explicit knowledge and skill building, overlooking potential adjustments in attitudes that could lead to more effective and permanent behavior change [36].

## Methods

Our study follows a convergent parallel, mixed methods design [45]. Quantitative data were collected using structured, self-administered, online surveys and qualitative data were collected through semi-structured interviews with administrators and focus groups with staff. A detailed protocol describing our guiding theoretical framework and methods has been previously published [26].

### Setting and participants

We purposefully selected three organizations for study participation based on key differences to ensure findings were related to the implementation strategy, rather than structural or organizational-level similarities [46, 47].^1^ Administrators at each organization required all staff affiliated with the program undergoing implementation to participate in HFTAT activities as appropriate for their position, though participation in research activities was voluntary. In total, one hundred and thirteen individuals participated in the study. Table 1 displays key differences in the programs and the number of individuals who participated in each type of data collection by organization.

**Table 1:** Characteristics and dates of engagement for organizations participating in HFTAT testing

	Organization 1	Organization 2	Organization 3
Location	Indianapolis, IN	Chicago, IL suburbs	Cincinnati, OH
Tenant population size	< 50	200	375
Years in operation	0	49	25
Housing model at time of HFTAT engagement	None because new program	Abstinence-only	Housing First
Housing type^a^	Project-based	Scattered-site	Scattered-site
Dates of HFTAT engagement	Nov 2015-June 2016	March 2016-Oct 2016	Sept 2016-March 2017
Number participating in each stage of data collection			
Online data collection	18	46	49
Administrative interviews	4	5	6
Staff focus group participants	4	6	2^b^

^a^“Project-based” refers to a program where all housing units are in a single building. “Scattered-site” refers to a program where housing units are scattered among multiple buildings

^b^While Org3 contracted multiple organizations to provide mental health case management, the organization only employed two housing case managers

### Measures and procedure

Specific implementation outcomes of focus in this paper include: (1) satisfaction with the HFTAT; (2) participant learning/knowledge resulting from eLearning; (3) acceptability and (4) appropriateness of the HF model, and (5) HF model fidelity. We measured HFTAT satisfaction with the 12-item Training Satisfaction Rating Scale [48], using two modified versions to account for: (a) training/eLearning satisfaction delivered to each participant at the time they completed the all modules and (b) TA satisfaction delivered to the implementation team members at the end of consultation activities. Participant learning/knowledge was measured using a 16-item, multiple choice test of eLearning content developed by the researchers, which was delivered to each participant at the immediate end of training and again 3 months after the end of consultation activities to measure knowledge retention. Performance was assessed based on percent of questions participants answered correctly. We measured acceptability using the Evidence-Based Practice Attitudes Scale [49], which has 15 items that ask respondents to rate their attitudes toward adoption of a new EBI. This scale was administered at baseline (i.e., before eLearning activates), at the immediate end of eLearning, and three months after the end of consultation activities. We measured fidelity using the HF Model Fidelity Index [26], which seeks to understand the level of implementation for 29 defined elements of the HF model. Fidelity data were collected as part of an audit and feedback process conducted by the TA providers, and were collected at Baseline, 3 months, 6 months, and 9 months.^2^

We collected additional information related to each of these measures, as well as acceptability of the HF model, through semi-structured qualitative, in-person focus groups conducted with staff and individual telephone interviews conducted with members of the implementation team. Sessions ranged from 30 to 72 min, with focus groups taking longer to conduct (average time = 65 min) than individual interviews (average time = 38 min). These activities were completed by the investigator EQA and a trained research assistant.

### Analysis

Regarding the analyses of quantitative data, we calculated: descriptive statistics to summarize participant characteristics and measures, frequencies and percentages for categorical variables, and means and standard deviations for continuous variables (e.g., age and Likert-type scales). We ran Chi-Square analyses on key characteristics (e.g., age, gender, race, ethnicity, time with organization, and baseline scores on outcome measures) between those who contributed to all data collection activities and those who did not to determine any potential differences that could have influenced participation rates. We evaluated scale reliability using Cronbach’s Alpha. We examined changes in participant learning/knowledge and acceptability of the HF model (measured by the evidence-based practice attitude scores) using the linear mixed effects model because it provides a flexible framework for handling missing data and providing valid estimates when data were missing at random. Longitudinal measures of participant learning/knowledge and acceptability of the HF model at all time points, including baseline, were incorporated in the model as the dependent variable. Participant-specific random effects were used to accommodate the correlation of longitudinal measures. Time of measurement was considered as a categorical variable to allow the non-linear longitudinal trend. Analysis of fidelity focused on changes in program scores at each time point. Primary quantitative analyses were performed using SAS 9.4 software [50].

Qualitative analysis was primarily deductive [51, 52], focusing on overall themes, as well as differences between administrators and staff. The principal investigator [DPW] developed a provisional coding scheme comprising descriptive codes (e.g., technical assistance) and values codes (e.g., appropriateness) derived from the primary research questions and theoretical framework guiding the study [53]. The list included three major code groups related to the implementation strategy, causal factors, and outcomes, each of which had sub-codes to account for specific components of each. DPW and EQA discussed the code list and separately coded a subset of transcripts in MAXQDA qualitative data analysis software [54]. The two researchers discussed discrepancies in coding clarifying different coding choices, solidifying code definitions, and adding additional codes to reflect concepts not covered in the original coding scheme.

## Results

A total of 113 individuals completed baseline data collection. Their characteristics are summarized in Table 2. We found no significant differences between those who participated in data collection at different levels (detailed results not shown).

**Table 2:** Participant characteristics (*n* = 113)

	Value
Age, mean (SD)	37.3 (11.6)
Race	
White	81 (71.7%)
Black or African American	29 (25.7%)
Other	4 (3.6%)
Hispanic or Latino/a	
Yes	7 (6.2%)
No	104 (92%)
Unknown	2 (1.8%)
Female gender	87 (77%)
Position in agency	
Administration/Management	35 (30.7%)
Staff	79 (69.3%)
Case manager/worker	40 (51%)
Service/Housing coordinator	14 (18%)
Other^a^	25 (32%)

^a^Job titles provided were too varied to group in a meaningful way

### Satisfaction with the implementation strategy

As demonstrated in Table 3, satisfaction with both training (*n* = 91) and consultation (*n* = 20) was high across all domains of the Training Satisfaction Rating Scale, with average satisfaction scores above 4 on the 1/“totally disagree” to 5/“totally agree” Likert scale. Qualitative data reinforced quantitative ratings, while also providing useful critiques. The overall consensus of administrators and staff demonstrated they felt the HFTAT was a useful implementation strategy and both the training and consultation were necessary.

I do like the combination of the modules and the technical assistance [i.e., consultation]. I feel like it’s definitely a one-two-punch that’s needed…I feel like giving everybody a base to learn from those modules and to get an understanding…Then, the technical assistance and then the real-world examples, I feel like is what’s really gonna make the difference. (Org3 Administrator)

Several participants also specifically stated the combined approach pushed their organizations to modify policies and procedures key to HF service delivery, including: removing HF-inconsistent language from official documents; changing job descriptions for better HF-alignment in roles; and changing processes for settling tenant-landlord disputes.

**Table 3:** Participant satisfaction with eLearning and technical assistance

	Training Activities^a^ (*n* = 91)		Consultation Activities^b^ (*n* = 20)	
	mean (SD)	Chronbach’s Alpha	mean (SD)	Chronbach’s Alpha
Overall score	4.04 (0.55)	0.92	4.12 (0.53)	0.95
Objective and content	4.09 (0.68)	0.88	4.07 (0.55)	0.83
Method and training context	3.94 (0.54)	0.83	4.10 (0.57)	0.92
Usefulness and overall rating	4.09 (0.63)	0.81	4.20 (0.56)	0.81

*All questions measured using a 1–5 Likert-type scale

^a^Questions administered to all individuals involved in HFTAT activities

^b^Questions only asked of individuals engaged in technical assistance activities

In the rest of this section, we provide a breakdown of participant discussions of satisfaction with components of the HFTAT. We only highlight those components that were discussed at enough length to develop clear qualitative themes related to them.

#### eLearning modules

Specific to the HFTAT’s training component, both staff and administrators discussed the eLearning modules’ compartmentalized, self-paced, interactive nature as particular strengths, with some individuals comparing it to what they described as fatiguing, all-day, in-person trainings they had taken in the past: “I liked that it was broken down into different modules, so that way you weren’t sitting there trying to watch something for eight hours, all day” (Org2 Administrator). They also described the narrative stories, particularly those of clients, as ringing true:

They [the stories] seemed realistic. They seemed pretty typical of clients we might see…I think that sort of stuff is helpful because it just makes you see this is not just some idealistic case…I hear case examples used in trainings, it’s like here’s the scenario and here’s what we did and why it worked out perfectly in the end, and it's like a storybook. And our clients are, they're complicated…so it's nice to hear cases, where you can relate to them. (Org2 Administrator)

As demonstrated in the comment above, the stories seemed more relatable to participants because they focused on both positive and negative situations and outcomes, thus reflecting the reality of their actual work.

Participants also discussed how the training helped facilitate conversations in their organizations, leading to a change in the way they approached problems: “…[b]ringing the HF modules, and this program specifically [to our organization]…has made it [HF] more part of th[e] discussion on an everyday basis [when we are dealing with issues], so that we’re problem-solving…we are being more empathetic about housing as a right versus a privilege and client choice...” (Org3 Staff).

Despite overall satisfaction, administrators discussed how the modules were necessary but not sufficient for facilitating HF implementation. They also discussed having to push a few staff to complete the modules. One clear theme among staff was they wished the modules had more information regarding how to work with situations that occur in the community, particularly how to deal with landlords, what happens to tenants after they are housed, how to help tenants meet their goals, and staff safety.

#### Digital badges

Feelings regarding the digital badges were mixed, with some participants stating they were not useful motivators, declaring instead that a displayable certificate would be better. Others stated they liked the badges because they demonstrated training completion to supervisors. Still, there were others who needed to be reminded what the badges were or who said they did not really understand their purpose: “I’m not sure what it [digital badges] could mean for me professionally…So, I was just like, ok, cool, I have proof of the trainings that I’ve completed, and that’s the way I looked at it and left it” (Org1 Staff). While this comment demonstrates the general lack of value participants found in the digital badges, another comment from an administrator demonstrates the value of the badges might also be depreciated for those who are not engaged in social media: “I don’t remember those [digital badges] at all, but I’m also not on any [sort] of social media” (Org3 Administrator).

#### Community of practice

There were very few in-depth discussions of the CoP, with most happening in administrative interviews. Generally, administrators stated they did not use the CoP, with reasons for this including not understanding how to access it, forgetting about it, or feeling it was unnecessary because they did not engage in front line work with tenants. Instead, some suggested supervisors might remind direct service staff to use it as a resource when they work with clients.

[Supervisors could use the CoP] [t]o connect that to whatever may be a challenge for the staff member, and say, "Well, when this is something that's a challenge for you, maybe you could go to this resource that's really helpful." (Org1 Staff)

In addition to our qualitative data, when we reviewed the CoP forum, discussions demonstrated low levels of engagement with an average of only 5 comments per each thread that participants were encouraged to contribute to.

#### Technical assistance calls

Discussions regarding TA occurred in administrator interviews, as the majority of staff were not engaged in these activities. Administrators stated the weekly TA phone calls helped them apply what they learned in the modules to specific cases in their organizations, and this aspect of the HFTAT was necessary to understand what HF truly means in practice:

…in terms of the whole harm reduction and everything that HF encompasses, [participating in the HFTAT] has helped us take different approaches, maybe what we wouldn’t have done before…I just remember one time we were processing something on that weekly call, and [the TA provider] had said, why don’t you try this? And it totally caught me off-guard, because, again, when I think of harm reduction, I just think of substance use, but it’s not just substance use, it’s harm reduction in every aspect. (Org1 Administrator)

While this participant understood the concept of harm reduction as it relates to substance use, working through tenant issues during TA helped them understand how the concept could be applied to a wide range of issues encountered in HF practice.

Administrators also appreciated the expertise and depth of the consultants. Other positive points of the TA discussed included: feeling supported with challenges, forcing conversations with agency partners, and assisting with HF policy and procedure refinement and development: “you want somebody [providing TA] that presents as knowledgeable and you can tell has experience in the field…I felt confident in that with them” (Org3 Administrator).

Regarding critiques, there were some sentiments the TA calls were too frequent and lasted for too many months, as in the case of one Org2 Administrator who stated the calls “could have been shortened a little bit” since their staff were already adept at delivering housing services. Though, other discussions indicated consultation should have been longer to include more “real-world examples” (Org3 Administrator) and walk people through how to best approach them. A few people also stated the TA could be expanded to include more staff.

#### Fidelity audit and feedback

Administrators discussed how they found the audit and feedback process useful as a conversation starter regarding particular HF elements they could improve on:

…[T]here were a few elements that I think were more surprising…things they didn’t really realize were necessarily part of HF…It was an opportunity for quite a conversation around maybe elements of HF that we should look at implementing better…. (Org3 Administrator)

Staff generally did not know how information gained through fidelity reviews with consultants was used, which is not surprising considering they did not participate in these activities.

### Housing first model knowledge

A primary goal of the eLearning modules was to improve participants’ HF knowledge prior to their engagement in implementation activities. As shown in Table 4, average quiz scores demonstrate HF knowledge was high among participants immediately after completing training, where the percent of questions correctly answered averaged 92% (SD = 31%). Demonstrating knowledge retention, average scores did not significantly change when quizzes were administered again three months after training ended (*p* = 0.19), and in fact increased to 98% (SD = 21%).

**Table 4:** Knowledge acquisition and retention

	End of training (*n* = 91)	3-month follow-up (*n* = 58)	Difference between time points^a^ (*n* = 58)	
	mean (SD)	mean (SD)	mean (SE)	*p*-value
Overall score	0.92 (0.310)	0.98 (0.21)	0.04 (0.03)	0.19

^a^Calculated using mixed-effects model

Discussions with administrators and staff demonstrated the value of knowledge gained through the training, such as one staff person who stated, “I’ve come a long way with just the total understanding of the program [i.e., HF model]” (Org1 Staff). Another person mentioned having learned much more about HF, even when they thought they already understood it pretty well:

I just am extremely grateful for all the training and technical assistance that has been given…I had a very simple understanding of HF. I thought really HF was just about, oh, encouraging people to reduce their usage of their drug[s] or alcohol. (Org1 Administrator)

Staff also discussed how the modules helped them understand the importance of client choice, and they demonstrated their knowledge through the strong descriptions of the HF model they provided:

…[HF] focuses on getting them [tenants] to be stable in their housing and learning independent living skills without focusing so much on drug use or certain behaviors. So, that once they are established with all of that [stability and living skills], then they could work on what they need to work on. (Org 2 staff)

Administrator discussions demonstrated how knowledge gained helped them connect HF to the bigger picture in terms of how the model related to the larger system, as in the case of one administrator who said the training helped them understand “why HUD [U.S. Department of Housing and Urban Development] wants all of their agencies to be HF” (Org3 Administrator) because they now understood prioritizing the most vulnerable members of the homeless population was a more effective strategy than casting a wide net of services.

### Acceptability and appropriateness of the HF intervention

Table 5 shows average overall and sub-dimension scores on the Evidence-Based Practice Attitudes Scale for participants at all three data collection points. As demonstrated, overall attitudes toward evidence-based practices were generally high at baseline. No significant changes in average overall score or sub-dimension scores were observed between baseline and either of the two follow-up points. As high scores at baseline could have prevented meaningful changes in those participants who scored lower from being observed, we conducted a second analysis focusing on those who scored below a 4 at baseline. As with the first analysis, average satisfaction scores were high. For this lower scoring group of participants, significant improvements between baseline and end of training were found for the overall score (*p* ≤ 0.01) and the requirements (p ≤ 0.01), appeal (p ≤ 0.01), and openness (*p* ≤ 0.0001) subscales. While some improvement in the requirements and appeal subscales was seen at the end of technical assistance, change in overall score was negligible at that time point.

**Table 5:** Changes in participant attitudes toward evidence-based practices

	T-1 Baseline		T-2 End of training		T-3 End of technical assistance		Difference T1 & T2		Difference T1 & T3^a^	
	n	mean (SD)	n	mean (SD)	n	mean (SD)	n	mean (SE)	n	mean (SE)
All participants										
Overall score	113	3.47 (0.63)	91	3.53 (0.55)	51	3.52 (0.44)	91	0.03 (0.05)	51	0.04 (0.07)
Requirements subscale		3.97 (1.04)		4.03 (0.90)		4.02 (0.91)		0.03 (0.09)		0.10 (0.12)
Appeal subscale		3.91 (0.90)		3.95 (0.75)		4.06 (0.58)		0.01 (0.08)		0.10 (0.12)
Openness subscale		3.82 (0.79)		3.98 (0.73)		3.86 (0.70)		0.12 (0.07)		−0.02 (0.09)
Divergence subscale		2.19 (0.74)		2.17 (0.91)		2.16 (0.75)		−0.02 (0.08)		−0.01 (0.10)
Participant baseline score less than 4										
Overall score	93	3.32 (0.59)	75	3.47 (0.55)	44	3.47 (0.43)	75	0.12 (0.06)*	44	0.11 (0.07)
Requirements subscale	66	3.30 (0.85)	53	3.68 (0.86)	31	3.69 (0.89)	53	0.33 (0.12)**	31	0.43 (0.15)**
Appeal subscale	65	3.37 (0.81)	50	3.75 (0.75)	28	3.88 (0.46)	50	0.35 (0.11)**	28	0.46 (0.14)***
Openness subscale	76	3.42 (0.62)	59	3.80 (0.75)	33	3.65 (0.69)	59	0.35 (0.09)****	33	0.17 (0.11)
Divergence subscale	112	2.17 (0.71)	89	2.17 (0.91)	50	2.16 (0.76)	89	0.01 (0.08)	50	−0.01 (0.10)

All questions pertaining to attitudes were measured using a 1–4 Likert-type scale, with higher scores indicating more accepting attitudes of evidence-based practices

^a^Calculated using mixed-effects model

*p ≤ 0.05; **p ≤ 0.01; ***p ≤ 0.001; ****p ≤ 0.0001

Qualitative data assisted us in understanding the extent to which the HF intervention itself (rather than EBIs in general) was understood to be both acceptable and appropriate to participants. The general feeling among participants was that HF was an acceptable intervention for clients. Administrators described HF as a holistic approach that utilized harm reduction to focus on “stages of change” and finding “small victories” such as ensuring tenants have security and food access. One administrator from Org2, previously an abstinence-only organization, discussed how they felt engagement in the HFTAT had impacted staff attitudes toward the model and new client population they were serving:

I don’t wanna say that staff would have been intolerant of clients prior [to engagement in the HFTAT], but I think that just learning more about HF…I think that’s kind of widened staff’s eyes a little bit…then, it kind of trickles down to just being a little bit more tolerant of sometimes when the day isn’t going your way." (Org2 Administrator)

Discussions with staff pointed toward a number of what they saw as positive aspects of HF, including: eliminating barriers to address immediate housing needs; expanding the population served; not having to force people to change; showing staff how to be more tolerant; and requiring more creativity from staff to carry out.

Despite training, a small number of staff did have issues with the harm reduction component of the intervention, stating harm reduction could enable active users of illicit substances or bringing up concerns regarding their personal safety in relation to clients with difficult-to-manage behaviors.

There were times that I questioned it because I’m just like “are we really implementing HF and harm reduction?”…I’m saying this because I had a lot of issues with safety concerns, for myself personally with some of the things that the tenants would do, and the way they would act and then our response to that. (Org1 Staff)

This particular issue was reinforced by administrators who expressed concerns that longer-term staff were having more difficulty with harm reduction component of the model; however, they also discussed the utility of the HFTAT in assisting staff to take a different approach when working with tenants, as in the case of an Org3 Administrator who felt the HFTAT provided a process sufficient enough in length and with enough real-world examples to help staff who were “entrenched in the accountability [i.e., abstience-focused] model” to begin to think about tenants and their problems differently.

Regarding appropriateness of the intervention, participants generally viewed HF as compatible with their existing organizational cultures, which emphasized “meeting people where they are”. However, they did note a number of incompatibilities that had to be overcome including the need for smaller caseloads given complexities of new tenants; more intensive work needed to preserve landlord relationships; incompatibilities between necessary HF services and their current billing structures; and concerns about the sobriety of tenants who have been in recovery. However, these discussions also showed the organizations had made or were making plans to address these issues based on TA provided: “the [TA] calls have helped a lot…especially with policies and procedures and stuff like that, actual forms that could be used as a template so we don’t have to reinvent the wheel.” (Org1 Staff).

#### Housing first model fidelity

**Fig. 1:**
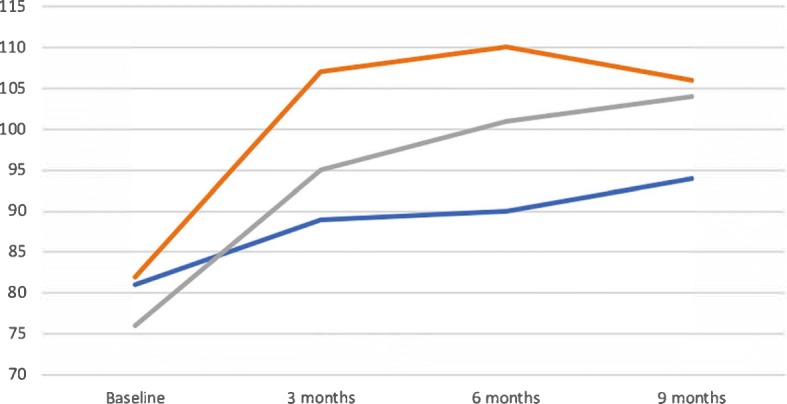
Changes in program’s fidelity scores over time

Regarding fidelity, 80 reflects the lowest score for a “true” HF program should receive on the instrument, and all programs started slightly below or slightly above this score at baseline, with scores ranging from 76 (Org3) and 82 (Org2). As shown in Fig. 1, all three programs saw improvements that ranged between 13 (Org1) and 28 (Org3) points, with an average improvement of 21.67 points among all programs. While Org1 and Org3 made improvements at all time points, Org2’s score reduced slightly from 110 to 106 between 6- and 9-month fidelity reviews (this is because the review at this time point evidenced new concerns regarding program termination guidelines that had not been identified previously).

## Discussion

This study supports previous research pointing to the value of combined training and consultation within an implementation strategy [1, 24, 41, 42], while also providing insights regarding a number of other strategies nested within the HFTAT’s two main components (e.g., narrative storytelling, CoP, digital badging, fidelity audit and feedback). Overall, training was useful in that it gave all participants baseline knowledge to prepare them to work in a HF program, while consultation activities provided implementation team members with support necessary to lead the implementation effort through TA and audit and feedback activities that developed their problem-solving skills and led to structural changes needed to improve fidelity. As such, the findings also support the usefulness of multifaceted strategies as an approach to implementing complex EBIs that can effectively engage multiple levels of an organization over a sustained period of time (6 months in this case) [6, 22, 23].

As demonstrated by the high satisfaction scores, both training and consultation arms of the HFTAT were well-liked by participants. Focusing on the training, the components that seemed to be responsible for participants’ positive reception of the modules included the flexibility inherent in the asynchronous online approach, the high level of interactivity, and the narrative stories. These three components resulted in a training that (1) was compatible with participants’ workflow; (2) kept their attention; and (3) presented information that fit their prior experience. Previous work looking at the implementation process has demonstrated the fit between what (a) workers are asked to do and learn and (b) their current work environment and past experiences are important predictors of implementation success [49, 55–57]. This also helps explain the lack of interest in the digital badges among participants, as they did not fit with current credentialing or continuing education practices that were meaningful to them [58–60].

We only gained a partial understanding of participants’ lack of interest in the CoP since it was discussed among administrators more than staff. Our previous work focused on the development of the eLearning modules provides more insight than the current data allow [27]. In this preliminary work with a different sample, we found some evidence that, like digital badging, incompatibility between the current work setting and the CoP was partially responsible for lack of engagement. We also found a lack of trust in the security of the online environment that made participants uneasy when interacting with it. This is a significant issue, as trust between members is considered a key component of a successful CoP [36]. Ponsford et al. [39] found both of these factors to be a barrier to success of an online learning community for alcohol harm reduction, as they noted study participants preferred to seek out known and trusted sources of information and a lack of fit between CoP access and participants’ daily work routines. Lack of interest in the HFTAT’s online CoP is not a total loss since discussions demonstrated eLearning facilitated discussions among participants that were reflective of a developing culture of learning. As such, it might be a more fruitful endeavor to foster the development of an internal CoP to connect and expand on isolated conversations that are happening. Having the implementation team lead this endeavor could help ensure alignment between organizational leadership’s expectations, agency resources, and staff attitudes and morale [21]. In the case of the HFTAT, an internal CoP might have improved knowledge transfer between the implementation team and staff members, thus giving staff more insight into how to deal with situations specific to their organization, which focus groups demonstrated they desired.

One of the HFTAT’s greatest successes is the observed positive change in attitudes toward EBI’s and harm reduction. There is existing evidence that the way EBIs are presented to professionals can impact attitudes toward them [57, 61], and our findings provide support for narrative storytelling as an approach for introducing effective but controversial practices to potentially resistant individuals. Qualitative data demonstrated that much of the success of this approach is because the stories reflected HFTAT participants’ experiences (demonstrating both successes and challenges), and changes in scores on the Evidence-Based Practice Attitudes scale suggest this attitude shift may have extended beyond the HF model to EBIs in general. A number of aspects HFTAT participants discussed liking (e.g., holistic approach, focusing on small changes, client-centeredness) about HF are reflective of its recovery-oriented approach. This is not surprising considering prior research on HF specifically and mental health services more generally has demonstrated staff working in environments they perceive as supportive of a recovery-orientation leads to greater satisfaction with their work [62, 63].

Whereas most previous research looking at training and/or consultation has focused on individual-level fidelity to clinical practices, this study demonstrates the benefit of these approaches for improving structural-level fidelity. All programs improved in fidelity over time. While programs did not reach 100 % fidelity, this was not the goal of the HFTAT. Indeed, there is general agreement in the implementation literature that adaptations are allowable as long as they are ‘fidelity consistent’ (i.e., do not modify the critical components demonstrated to impact desired outcomes) [64, 65], and previous research has demonstrated flexibility in HF fidelity during the implementation process is important due to the significant influence the local community can have [66]. Instead, consultants worked with organizations to obtain as strong a fidelity score as was possible given contextual constraints, and implementation team members seemed to appreciate this approach based on the reflective conversations it initiated.

The mixed method approach was a particular strength of this study, as it demonstrated the HFTAT’s influence on key outcomes beyond what could have been gathered using a single approach. While not limited to this example, use of both quantitative and qualitative data had a particular advantage in relation to the measurement of participant attitudes. While use of a standardized measure provided results reflecting general EBI attitudes that can be easily compared with those from other studies, qualitative findings offered HF-specific information that is more useful for guiding future work in this particular area [57]. Additionally, triangulation of results from different methods and respondent types (e.g., staff and administrators) enhances validity and reliability of the study findings [45, 67]. For instance, qualitative themes related to participant knowledge enhance our confidence quantitative outcomes reflected actual learning, rather than the ease of test questions. Triangulation also lends support to the conclusion that significant positive changes in EBI attitudes observed after removing high scorers from the analysis were likely due to actual improvements rather than regression to the mean among the lowest scoring participants. While our focus on three organizations limits generalizability of the findings, selection of sites based on key contextual and organizational differences does increase the likelihood the findings are applicable to a variety of settings [46, 47]. A comparison of specific organizational characteristics (see Table 1) and their impact on the implementation process could lead to greater explanatory insights regarding the HFTAT’s ability to overcome specific implementation barriers. While this is beyond the scope of the current paper, we plan to address this issue in subsequent work. Finally, though the linear mixed model assumes individual-level follow-up data were missing at random due to factors such as turnover and high job demands characteristic of the work of housing staff, there is potential estimates were biased if this was not the case.

## Conclusion

All three organizations involved in the HFTAT improved along a variety of individual-level outcomes (e.g., satisfaction, acceptability and appropriateness, and HF knowledge) and structural-level fidelity, which supports prior research pointing to the value of both (a) multifaceted strategies and (b) combined training and consultation approaches. The need for compatibility between specific elements of and implementation strategy and the work environment was also observed, and lack of such compatibility likely explains the poor reception related to the CoP and digital badging components of the HFTAT. Most importantly as it relates to the HF model and other interventions employing evidence-based but controversial interventions, this study demonstrates the value of a narrative storytelling approach for changing attitudes that can act as a barrier to successful implementation. Future research on the HFTAT, and on multifaceted implementation strategies in general, should seek to understand the relative value of different components of the implementation strategy. Such an understanding could lead to more streamlined and cost-effective approaches should results support dropping less effective components.

## Additional file

Additional file 1: HFTAT Specifications. This file contains a more detailed description of the HFTAT’s components mapped onto recommended implementation strategy description guidelines. (PDF 71 kb)

## Abbreviations

CoP: Community of practice
EBI: Evidence-based intervention
HF: Housing First
HFTAT: Housing First Technical Assistance and Training Strategy
Org1: Organization 1
Org2: Organization 2
Org3: Organization 3
TA: Technical assistance

## Footnotes

1 This is a strategic deviation from our original protocol’s goal to recruit 4 organizations between two cities. The change is due to unplanned opportunities presented during the course of the study: Org1 was a brand-new program without a pre-existing service model that required significantly more up-front time and resources and Org 2 reached out to us upon learning about the study, presenting an opportunity to work with a program outside our original target states. Therefore, the final decision to reduce our sample size was based on the unexpected diversity these two programs brought to the sample and their ability to add significant insights beyond our original expectations.

2 The 9-month timeframe for data collection is shorter than the 12 months listed in our original protocol. The reason for this adjustment was due to the rightly anticipated complexities of working with Org1 and Org3, which shortened the available data collection time due to the considerable front-end effort required during the engagement period.
