# Supplementary material for: The housing first technical assistance and training (HFTAT) implementation strategy: outcomes from a mixed methods study of three programs
Source: Subst Abuse Treat Prev Policy. 2018 Sep 21;13:32. doi: 10.1186/s13011-018-0172-3 (PMC6151066; doi:10.1186/s13011-018-0172-3)
Supplement: Supplementary file 1 — HFTAT Specifications. This file contains a more detailed description of the HFTAT’s components mapped onto recommended implementation strategy description guidelines. (PDF 71 kb) [file 13011_2018_172_MOESM1_ESM.pdf]

Additional file 1

The table below describes the HFTAT following Proctor et al's [1] recommendations for specifying and reporting implementation strategies. We also utilize Powell et al's [2] refined taxonomy of implementation strategies to ensure consistency of the HFTAT's description with prevailing views in the field. However, we do differ from Powell et al. in that we view their taxonomy as listing activities one must carry out when executing an implementaiton strategy, rather than unique implementation strategies in themselves [see 3]. As such, we present what we consider to be the individual strategies making up the HFTAT under "Defined components" and present Powell et al's strategies under "Actions".

**Specifications of the Housing First Technical Assistance and Training (HFTAT) program: A multifaceted and nested strategy to guide Housing First implementation**		
**Defined components**		
Primary components	eLearning-based Training	Distance-based consultation
Nested components	• Interactive activities<br>• Opportunities for reflection<br>• Narrative story telling<br>• Community of practice<br>• Digital badging	• Implementation manual<br>• Baseline assessment<br>• Tailored implementation planning<br>• Technical assistance<br>• Fidelity audit and feedback
**Domains of specification**[2]		
Actor	Pre-recorded training developed academics and practitioners with expertise in Housing First and eLearning	Expert Housing First practitioners
Actions[1]	• Conduct ongoing training<br>• Create a learning collaborative<br>• Create or change credentialing<br>• Develop educational materials<br>• Distribute educational materials<br>• Make training dynamic	• Assess for readiness and identify barriers and facilitators<br>• Audit and provide feedback<br>• Centralize technical assistance<br>• Develop a formal implementation blueprint<br>• Facilitation<br>• Identify and prepare champions<br>• Mandate change

		• Obtain formal commitments<br>• Promote adaptability<br>• Provide ongoing consultation<br>• Train leadership<br>• Tailor strategies
Action targets	• Knowledge<br>• Skills<br>• Attitudes	• Implementation leadership skills<br>• Improved problem solving/troubleshooting in harm reduction environment<br>• Development/refinement of policies and procedures<br>• Appropriate adaptation of intervention to local context
Temporality	Differs by role in organization<br>• Training for administrators and other members of implementation team should be completed 1 week before technical assistance begins.<br>• Staff training begins no sooner than one week after first technical assistance meeting but can wait until implementation team feels comfortable for implementation activities to begin.<br>• Training should take no longer than 1 month. New staff to agency can engage training at any point.	First session within one week of end-of-training deadline and one month before implementation activities begin.
Dose	Four modules (1-2 hours each)<br>• Administrators take all four modules	One hour once a week for six months. Sessions extended if a week is missed.

	• Clinical and case management staff take modules 1-3<br>• All other staff take module 1	
Implementation outcome affected	• Acceptability and appropriateness<br>• Adoption of necessary practices and skills to work in harm reduction environment<br>• Introduction to necessary structural changes and management practices to improve fidelity and sustainability	• Acceptability of the intervention<br>• Program fidelity<br>• Sustainability
Justification	• eLearning is scalable and cost effective, and addresses staff turnover<br>• Research suggests educational approaches may help overcome attitudinal barriers to harm reduction noted in literature<br>• Incentives may enhance staff motivation	• Need to develop strong leadership in light of Housing First's noted complexity<br>• Prior research shows Housing First adaptations are often necessary

[1]Refers to implementation strategies defined by Powell et al. [2] that are reflected in the HFTAT's components and/or activities associated with them.
[2]Domains of implementation strategy reporting as defined by Proctor et al. [1].

References

1. Proctor EK, Powell BJ, McMillen JC. Implementation strategies: recommendations for specifying and reporting. Implement Sci. 2013;8:139.

2. Powell BJ, Waltz TJ, Damschroder LJ, Smith JL, Matthieu MM, Proctor EK, et al. A refined compilation of implementation strategies: results from the Expert Recommendations for Implementing Change (ERIC) project. Implement Sci. 2015;10:21.

3. Saldana L. The stages of implementation completion for evidence-based practice: protocol for a mixed methods study. Implement Sci. 2014;9:43.
